# Comparative cytogenetics of anembryonic pregnancies and missed abortions in human

**DOI:** 10.18699/VJGB-23-05

**Published:** 2023-03

**Authors:** T.V. Nikitina, E.A. Sazhenova, E.N. Tolmacheva,, N.N. Sukhanova,, S.A. Vasilyev,, I.N. Lebedev

**Affiliations:** Research Institute of Medical Genetics, Tomsk National Research Medical Center of the Russian Academy of Sciences, Tomsk, Russia; Research Institute of Medical Genetics, Tomsk National Research Medical Center of the Russian Academy of Sciences, Tomsk, Russia; Research Institute of Medical Genetics, Tomsk National Research Medical Center of the Russian Academy of Sciences, Tomsk, Russia; Research Institute of Medical Genetics, Tomsk National Research Medical Center of the Russian Academy of Sciences, Tomsk, Russia; Research Institute of Medical Genetics, Tomsk National Research Medical Center of the Russian Academy of Sciences, Tomsk, Russia; Research Institute of Medical Genetics, Tomsk National Research Medical Center of the Russian Academy of Sciences, Tomsk, Russia

**Keywords:** anembryonic pregnancy, missed abortion, miscarriage, karyotype, chromosomal abnormalities, sex chromosomes, triploidy, tetraploidy, анэмбриония, неразвивающаяся беременность, невынашивание беременности, кариотип, хромосомные аномалии, половые хромосомы, триплоидия, тетраплоидия

## Abstract

Miscarriage is an important problem in human reproduction, affecting 10–15 % of clinically recognized pregnancies. The cases of embryonic death can be divided into missed abortion (MA), for which the ultrasound sign of the embryo death is the absence of cardiac activity, and anembryonic pregnancy (AP) without an embryo in the gestational sac. The aim of this study was to compare the frequency of chromosomal abnormalities in extraembryonic tissues detected by conventional cytogenetic analysis of spontaneous abortions depending on the presence or absence of an embryo. This is a retrospective study of 1551 spontaneous abortions analyzed using GTG-banding from 1990 to 2022 (266 cases of AP and 1285 cases of MA). A comparative analysis of the frequency of chromosomal abnormalities and the distribution of karyotype frequencies depending on the presence of an embryo in the gestational sac was carried out. Statistical analysis was performed using a chi-square test with a p <0.05 significance level. The total frequency of chromosomal abnormalities in the study was 53.6 % (832/1551). The proportion of abnormal karyotypes in the AP and MA groups did not differ significantly and amounted to 57.1 % (152/266) and 52.9 % (680/1285) for AP and MA, respectively (p = 0.209). Sex chromosome aneuploidies and triploidies were significantly less common in the AP group than in the MA group (2.3 % (6/266) vs 6.8 % (88/1285), p = 0.005 and 4.9 % (13/266) vs 8.9 % (114/1285), p = 0.031, respectively). Tetraploidies were registered more frequently in AP compared to MA (12.4 % (33/266) vs. 8.2 % (106/1285), p = 0.031). The sex ratio among abortions with a normal karyotype was 0.54 and 0.74 for AP and MA, respectively. Thus, although the frequencies of some types of chromosomal pathology differ between AP and MA, the total frequency of chromosomal abnormalities in AP is not increased compared to MA, which indicates the need to search for the causes of AP at other levels of the genome organization, including microstructural chromosomal rearrangements, monogenic mutations, imprinting disorders, and epigenetic abnormalities.

## Abstract (ru)

Невынашивание беременности является серьезной проблемой в репродукции человека, затрагивающей 10–15 % клинически распознаваемых беременностей. Среди случаев эмбриональной гибели можно выделить замершие (неразвивающиеся) беременности (НБ), при которых ультразвуковым признаком гибели эмбриона служит отсутствие сердцебиения, и анэмбрионии (АЭ) – отсутствие эмбриона в полости плодного мешка. Целью данного исследования было сравнение частоты хромосомных аномалий во внезародышевых тканях, выявляемых при стандартном цитогенетическом анализе материала спонтанных абортов, в зависимости от наличия или отсутствия эмбриона. Проведено ретроспективное исследование 1551 спонтанного абортуса, проанализированного с помощью стандартного цитогенетического исследования с 1990 по 2022 г. (266 случаев АЭ и 1285 случаев НБ) в НИИ медицинской генетики Томского НИМЦ. Выполнен сравнительный анализ частоты хромосомных аномалий и распределения частот кариотипов в зависимости от наличия эмбриона в полости плодного мешка. Статистический анализ проводили с использованием критерия хи-квадрат с уровнем значимости p <0.05. Суммарно частота хромосомных аномалий в исследованной выборке составила 53.6 % (832/1551). Доля аномальных кариотипов в группах АЭ и НБ значимо не различалась и составила 57.1 % (152/266) и 52.9 % (680/1285) для АЭ и НБ соответственно (p = 0.209). При НБ статистически значимо чаще встре- чались аномалии числа половых хромосом (6.8 % (88/1285) против 2.3 % (6/266), p = 0.005) и триплоидии (8.9 % (114/1285) против 4.9 % (13/266), p = 0.031). В то же время при отсутствии эмбриона статистически значимо чаще регистрировалась тетраплоидия (12.4 % (33/266) против 8.2 % (106/1285), p = 0.031). Соотношение полов (46,XY : 46,XX) среди абортусов с нормальным кариотипом составило 0.54 и 0.74 для АЭ и НБ соответственно. Таким образом, хотя частоты некоторых типов хромосомных аномалий различаются между АЭ и НБ, суммарная частота хромосомных аномалий при АЭ не повышена по сравнению с НБ, что свидетельствует о необходимости поиска причин АЭ на других уровнях организации генома, включая микроструктурные перестройки хромосом, моногенные мутации, нарушения импринтинга и аберрантные эпигенетические модификации

## Introduction

Miscarriage is one of the most common issues in human reproduction that results in embryonic or fetal death in 10 to 15 % of all clinically recognized pregnancies (Larsen et al., 2013). Cytogenetic studies reveal chromosomal abnormalities in 50–60 % of first trimester abortions (Menasha et al., 2005; van den Berg et al., 2012; Hardy et al., 2016; Soler et al., 2017; Wang et al., 2020; Wu et al., 2021), and in recent years, there has been an increasing amount of data about the association of miscarriage with copy number variations (CNV), gene mutations, methylation abnormalities and other epigenetic aberrations (Levy et al., 2014; Fu et al., 2018; Fan et al., 2020; Finley et al., 2022). Identification of embryo death causes is necessary to assess the miscarriage risk in subsequent pregnancies; in addition, uncovering a pathogenic factor is important for psychological condition of the couples.

Anembryonic pregnancy is the absence of an embryo in the gestational sac, and it is one of the earliest forms of miscarriage. In anembryonic pregnancy, a blastocyst is implanted into the uterine wall, a gestational sac is formed, but the embryo itself either does not develop initially, or its formation arrests at the earliest stages (no later than the 5th week of gestation), and then only extra-embryonic components of the conceptus continue to proliferate and grow.

As a rule, at around 6 weeks of gestation, the secondary yolk sac and the primary germ layers could be detected within the gestational sac by transvaginal ultrasound, and primitive cardiac tube could be detected during the 7th week. In early pregnancy loss there are several ultrasonography features: the absence of embryonic cardiac activity with a diameter of the gestational sac ≥25 mm, crown–rump length (CRL) ≥7 mm for a period of 6 weeks or more; the absence of an embryo and its cardiac activity 14 days after the detection of a gestational sac without a yolk sac; the absence of an embryo and its cardiac activity 11 days after the detection of a gestational sac with a yolk sac (Doubilet et al., 2013). Thus, ultrasound scanning makes it possible to differentiate two forms of early embryonic death: anembryonic pregnancy (AP) and missed abortion (MA). AP is diagnosed in the absence of an embryo and a secondary yolk sac in the cavity of the gestational sac, for a period of more than 7 weeks (Radzinsky et al., 2015); in addition, ultrasound criteria for AP are a gestational sac more than 13 mm without a yolk sac or more than 18 mm without an embryo. The absence of cardiac activity in the presence of an embryo is a sign of МА

There are terminological inconsistencies, which make it difficult to compare the results of studies implemented in different centers. The ICD-10 uses the terms ‘blighted ovum’ and ‘missed abortion’, accepted many years ago (Robinson, 1975), which do not quite represent the clinical features found using the ultrasound examination (Farquharson et al., 2005). The European Society of Human Reproduction and Embryology (ESHRE) special group has proposed the terms ‘anembryonic (empty sac) miscarriage’ for a gestational sac ≥8 mm in diameter and without a yolk sac or embryo; ‘yolk sac miscarriage’ for a gestational sac with a yolk sac, but without an embryo; ‘embryonic miscarriage’ with an embryonic CRL of at least 7 mm without cardiac activity (Kolte et al., 2015). Thus, the diagnosis of AP includes both an empty gestational sac (empty sac) and a gestational sac with a yolk sac and without an embryo (yolk sac only).

The estimated frequency of AP among the first trimester pregnancy losses differs: from 16 % in early studies (Robinson, 1975), 22.6 % after IVF (Li et al., 2017), and up to 30–40 % in most studies (Lathi et al., 2007; Cheng et al., 2014; Ouyang et al., 2016; Yoneda et al., 2018). Despite the prevalence of AP, data on the frequency of chromosomal abnormalities in this pathology are contradictory. Intuitively, it seems that such early and pronounced violations, which lead to the developmental arrest of the embryo per se at the initial stages of its formation, should be associated with a significantly increased frequency and severity of chromosomal abnormalities. In some studies such association was found (Angiolucci et al., 2011). At the same time, most recent studies demonstrate either the absence of significant differences in the frequency of chromosomal abnormalities between the AP and MA groups (Lathi et al., 2007; Muñoz et al., 2010; Ljunger et al., 2011; Liu et al., 2015), or even a lower frequency of abnormal karyotypes in AP compared to MA (Ginsberg et al., 2001; Cheng et al., 2014; Li et al., 2017; Yoneda et al., 2018; Gu et al., 2021). Therefore, we consider it of current interest to study large samples of AP and MA cases in comparison with the published data. In this work, we studied the frequency and spectrum of chromosomal anomalies detected by cytogenetic analysis of 1551 cases of early miscarriage, depending on the presence or absence of an embryo.

## Materials and methods

The object of this study was 1551 spontaneous abortions, karyotyped in the Cytogenetic Laboratory of the Research Institute of Medical Genetics of the Tomsk National Research Medical Center. Products of conception (POC) were obtained from gynecological clinics of Tomsk and Seversk, along with information regarding the patient’s age, woman’s obstetric and gynecological history, and the number and outcomes of her previous pregnancies. The study was approved by the Biomedical Ethics Committee of the Research Institute of Medical Genetics of the Tomsk National Research Medical Center, Protocol 10, Feb. 15, 2021. Informed consents were obtained from all patients.

In most cases, abortion karyotypes were established using conventional GTG banding after long-term extra-embryonic fibroblast culture (90.9 %, 1410 samples) or direct preparations of the chorionic villi (1.9 %, 29 samples). Conventional comparative genomic hybridization (CGH) (3.5 %, 54 samples) and interphase fluorescence in situ hybridization (FISH) with centromere-enumeration probes (3.7 %, 58 samples) were performed in cases where traditional cytogenetic analysis failed. AP were diagnosed by ultrasound examination and included 266 (17.2 %) abortions (with absence of an embryo in the cavity of the gestational sac for more than 7 weeks, a gestational sac more than 13 mm without a yolk sac or more than 18 mm without an embryo). The other 1285 abortions (82.8 %) with an embryo were assigned to the MA group (where an embryonic pole was identified without cardiac activity).

The POC material, usually represented by the fragments of the gestational sac, was delivered to the laboratory in sterile saline, thoroughly washed from blood, and separated from decidual tissues. Methods of embryonic cells culture, chromosome preparations, cytogenetic techniques, FISH and CGH were performed as described previously (Lebedev, Nikitina, 2013).

The calculation of the statistical significance of differences between frequencies was performed using the χ2 analyses; the normality of the distribution for quantitative indicators was checked using the Kolmogorov–Smirnov test; due to the differences from the normal distribution, comparisons between groups were performed using the nonparametric Mann–Whitney test. A significance level of p < 0.05 was applied for all tests. The sex ratio (SR) was calculated as the ratio of karyotypes 46,XY : 46,XX. Recurrent pregnancy loss (RPL) was defined as two or more consecutive miscarriages in a woman’s obstetric history.

The study was performed at the Core Medical Genomics Facility of the Tomsk National Research Medical Center (NRMC) of the Russian Academy of Sciences using the resources of the bio-collection “Biobank of the population of Northern Eurasia” of the Research Institute of Medical Genetics, Tomsk NRMC.

## Results

Table 1 shows the comparison of the demographic characteristics of the studied groups of abortions. The age of mothers and fathers, the number of woman’s pregnancies and spontaneous abortions, and the proportion of couples with RPL in a woman’s history did not differ significantly in the samples. However, the gestational age (both by the date of the last menstrual period and by ultrasound examination) in the AP group was significantly less than in the MA group.

**Table 1.**
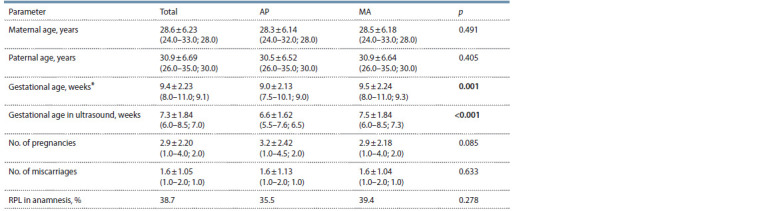
Comparison of demographic parameters of the AP and MA groups Mean ± standard deviation (lower and upper quartile; median); * gestational age calculated by the date of the last menstrual period. Significantly different rates are in bold

In total, abnormal karyotypes were found in 53.6 % (832/ 1551) of abortions. Table 2 shows the karyotype frequencies. The rates of the different types of chromosomal abnormalities among pregnancy losses with and without an embryo were 52.9 % (680/1285) and 57.1 % (152/266) respectively, and did not differ significantly ( p = 0.209). We found similar frequencies of chromosomal abnormalities between AP and MA in the autosomal trisomies (27.8 and 22.5 %), autosomal monosomies (1.5 and 0.6 %), structural aberrations (2.3 and 1.0 %) and combined anomalies that include combinations of different types of chromosomal aberrations in one abortion (4.9 and 4.2 %) (see Table 2). At the same time, numerical abnormalities of sex chromosomes in AP were three times less common than in MA (2.3 and 6.8 %, p = 0.005). This difference was even more pronounced for monosomy X: 0.8 % (2/266) and 5.0 % (64/1285), p < 0.001.

**Table 2.**
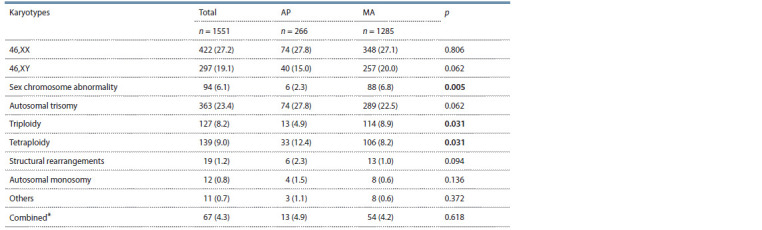
Karyotypes of abortions in the AP and MA groups Percentages are given in parentheses. * Combined – combination of different types of abnormalities. Significantly different rates are in bold.

Triploidies occurred significantly less frequently, and tetraploidies occurred significantly more frequently in abortions without embryo in comparison with embryonic miscarriages (4.9 and 8.9 %, p = 0.031; 12.4 and 8.2 %, p = 0.031 in AP and MA, respectively). Since some of the tetraploid karyotypes in mosaic form may represent cultural artifacts, we reexamined some tetraploid samples using FISH in non-cultured tissues. The frequency of FISH-confirmed tetraploidies showed even more statistically significant differences: 14/266 (5.3 %) at AP vs 22/1285 (1.7 %) in MA ( p < 0.001).

Among the abortions with normal karyotype, the SR was 0.54 for AP and 0.74 for MA; there were no significant differences in the distribution of 46,XX and 46,XY karyotypes (p = 0.142).

## Discussion

The aim of this study was to compare the frequency of chromosomal abnormalities in pregnancy losses with and without an embryo (MA and AP). In this study, we analyzed a large sample of miscarriages (1551 abortions) and did not find significant differences in the chromosomal abnormality rates between the AP and MA groups (57.1 and 52.9 % respectively, p = 0.209). An analysis of previously published comparative studies showed conflicting results regarding the correlation between the karyotype and the presence of an embryo in the gestational sac (Table 3).

**Table 3.**
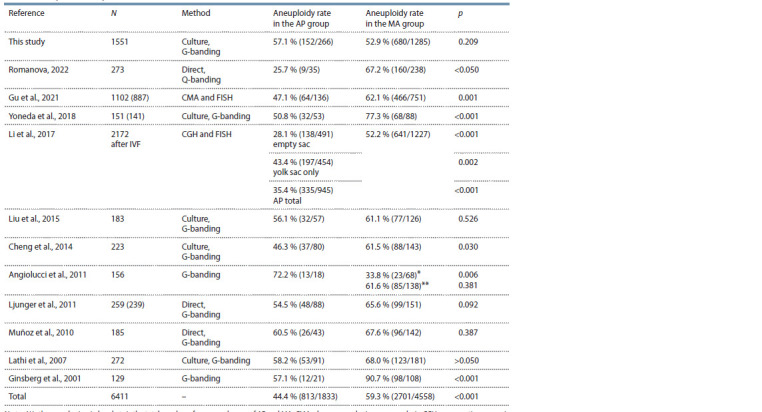
Comparative frequencies of chromosomal abnormalities in AP and MA in various studies N is the sample size, in brackets is the total number of compared cases of AP and MA; CMA, chromosomal microarray analysis; CGH, comparative genomic hybridization; FISH, fluorescence in situ hybridization. * Relative to abortions with a normal phenotype on ultrasound examination; ** relative to abortions with the presence of an embryo on ultrasound examination

The presence of a yolk sac in a gestational sac without an embryo means that the disorders appear after the segregation of the hypoblast and epiblast, which occurs 6–7 days after fertilization. The hypoblast, which gives rise to the endoderm of the yolk sac, continues to develop, while the epiblast, which gives rise to the three germ layers of the embryo itself (endoderm, ectoderm, and mesoderm), is blocked. An empty gestational sac means that the abnormalities appeared before the separation of the inner cell mass into hypoblast and epiblast, i. e. during implantation (Boss et al., 2018). At such an early stage, the influence of non-genetic factors is unlikely to be significant. The lower frequency of chromosomal abnormalities in such embryos may be due to the fact that at such early stages of development, damage of the activity of genes important in early embryogenesis due to point mutations, CNV, or epigenetic anomalies is more critical than a change in the gene dosage due to aneuploidy.

Considering the predominant contribution of genetic causes (compared to maternal or environmental causes) to a very early arrest of embryonic development, the lower frequency of chromosomal abnormalities in the absence of an embryo in the gestational sac makes it promising to search for genetic aberrations of the sub-chromosomal level and epigenetic anomalies in AP cases (Lebedev et al., 2013). Thus, chromosomal microarray analysis revealed a greater number of CNV in AP compared to MA (299 and 132, respectively), and in AP among pathogenic rearrangements 54.3 % deletions and 45.7 % duplications were found, whereas in MA only duplications were found (Savchenko et al., 2018). Interestingly, the set of genes in CNV also differed: in AP, the genes responsible for basic biological processes, such as migration, cell contacts, and adhesion, were more often affected, while in MA, the genes responsible for morphogenesis were affected.

We found different frequencies of some types of chromosomal abnormalities between abortions with and without an embryo. In our sample, sex chromosome aneuploidies (especially the 45,X) were less common in AP than in MA (see Table 2). Frequency of the 45,X karyotype has been found to be significantly higher in miscarriages with an embryo in most published comparative studies (Minelli et al., 1993; Muñoz et al., 2010; Cheng et al., 2014; Liu et al., 2015; Veropotvelyan, Kodunov, 2015; Li et al., 2017; Ozawa et al., 2019; Gu et al., 2021). These results indicate that monosomy X does not have a noticeable negative effect on the early development of the embryo per se, and such embryos die at later stages, possibly due to a failure of trophoblast function (Ahern et al., 2022).

Triploidy is another type of chromosomal abnormality, which is more common in embryonic miscarriages than in anembryonic ones. Previous studies suggested that the majority of triploidies are the result of fertilization errors leading to either diandry (the presence of two sets of paternal chromosomes) or digyny (the presence of two maternal sets) (Thaker, 2005). Due to the phenomenon of chromosomal imprinting, paternal chromosomes contribute to the preferential proliferation of trophoblast tissues. Perhaps it is diandric triploidy that leads to the AP phenotype, but this assumption needs to be verified.

Common feature for both of the above mentioned types of chromosomal abnormalities is that the mechanism of their origin is not associated with meiotic nondisjunction in oocytes. It is known that cases of X-chromosome monosomy are most often caused by errors in paternal meiosis (Hassold et al., 1988; Segawa et al., 2017), and triploidies are caused mostly by fertilization errors. Therefore, the rate of these types of karyotype abnormalities is increased among abortions from young mothers in comparison with older mothers (Soler et al., 2017; Wang et al., 2020; Gu et al., 2021). Since the mother’s age was similar in our AP and MA samples, the higher rate of monosomy X and triploidy in MA in comparison with AP supports the assumption that embryos with these karyotype abnormalities survive better.

We found a higher frequency of tetraploidy in the AP group, which is consistent with the data of (Veropotvelyan, Kodunov, 2015; Ozawa et al., 2019) and implies an unfavorable influence of the tetraploid karyotype, leading to an earlier termination of embryo development.

We found that sex ratio (SR) in abortions with normal karyotype deviates from the expected SR and constitutes 0.74 for MA and 0.54 for AP. Although the differences between the groups did not reach a statistically significant level ( p = 0.142), they are consistent with the data obtained earlier in our laboratory using significantly smaller samples. In the study (Evdokimova et al., 2000) it was shown that the proportion of 46,XY embryos inversely correlates with the severity of developmental disorders: the SR was 0.77 for spontaneous abortions without significant intrauterine delay of development; 0.60 for MA and 0.31 for AP (compared to 1.10 for control group of induced abortion). One of the reasons for the biased SR may be maternal cell contamination (MCC) of extra-embryonic cell cultures. But since both AP and MA samples were analyzed concomitantly, and the frequency of MCC was low (Nikitina et al., 2005), this equalizes the possible effect of maternal contamination on SR in our study. Interestingly, a large-scale study of SR in early human development (from conception to birth) showed that SR decreases in the first week after conception (due to excess male mortality) and then increases for at least 10–15 weeks (due to excess female mortality) (Orzack et al., 2019). Thus, the excess of female embryo loss in the first trimester of pregnancy probably represents a real phenomenon.

The development of cell-based technologies offers a unique opportunity to study the biological mechanisms that lead to embryogenesis failure. Thus, induced pluripotent stem cells (iPSCs) reproduce the characteristics of embryonic stem cells, including unlimited proliferative capacity and the ability to differentiate into derivatives of three germ layers (pluripotency). It has been shown that iPSCs can be derived from trophoblast tissues not only from embryos with a normal karyotype, but also from embryos with some chromosomal aneuploidies (for example, monosomy X and trisomy 13) (Parveen et al., 2017; Long et al., 2020). If it is possible to reprogram trophoblast cells and obtain iPSC lines from anembryonic cases, this will open up the possibility to study the processes in the derivatives of various germ layers leading to an early developmental arrest of the embryo.

## Conclusion

We found that the pattern of chromosomal abnormalities partly differs between AP and MA, and the presence of an embryo is positively correlated with sex chromosome aneuploidy and triploidy, while the absence of an embryo is positively correlated with tetraploidy. At the same time, the total frequency of chromosomal abnormalities in AP and MA did not differ, which indicates the need to search for the causes of AP at other levels of genome organization, including microstructural chromosomal rearrangements, monogenic mutations, imprinting disorders, and other aberrant epigenetic modifications of the genome.

## Conflict of interest

The authors declare no conflict of interest.
